# Metagenomics Assessment of Soil Fertilization on the Chemotaxis and Disease Suppressive Genes Abundance in the Maize Rhizosphere

**DOI:** 10.3390/genes12040535

**Published:** 2021-04-07

**Authors:** Matthew Chekwube Enebe, Olubukola Oluranti Babalola

**Affiliations:** Food Security and Safety Niche Area, Faculty of Natural and Agricultural Sciences, North-West University, Private Bag X2046, Mmabatho 2735, South Africa

**Keywords:** chemotaxis, disease suppressive soil, maize rhizosphere, metagenomes, soil fertilization

## Abstract

Soil fertility is a function of the level of organic and inorganic substances present in the soil, and it influences the activities of soil-borne microbes, plant growth performance and a host of other beneficial ecological functions. In this metagenomics study, we evaluated the response of maize microbial functional gene diversity involved in chemotaxis, antibiotics, siderophores, and antifungals producing genes within the rhizosphere of maize plants under compost, inorganic fertilizer, and unfertilized conditions. The results show that fertilization treatments at higher compost manure and lower inorganic fertilizer doses as well as maize plants itself in the unfertilized soil through rhizosphere effects share similar influences on the abundance of chemotaxis, siderophores, antifungal, and antibiotics synthesizing genes present in the samples, while higher doses of inorganic fertilizer and lower compost manure treatments significantly repress these genes. The implication is for a disease suppressive soil to be achieved, soil fertilization with high doses of compost manure fertilizer treatments as well as lower inorganic fertilizer should be used to enrich soil fertility and boost the abundance of chemotaxis and disease suppressive genes. Maize crops also should be planted sole or intercropped with other crops to enhance the rhizosphere effect of these plants in promoting the expression and abundance of these beneficial genes in the soil.

## 1. Introduction

The movement of microbes from one point in the environment to another as a result of potential differences in nutrients or proper chemical gradients is termed chemotaxis. Chemotaxis has many physiological roles, such as improving access to growth nutrients as well as in the initiation of infection. This movement is ATP (energy) dependent for efficient flagella and other locomotory structures of the microbes in responding to the chemical gradients stimuli. Chemotaxis is a survival mechanism of microbes either positively going towards or away from the source of the stimulus. The priming of the chemosensory pathways by the signaling molecules facilitates chemotaxis, and this begins when the chemical molecules bind to the receptors, which form complexes with *cheA* (a histidine kinase) and *cheW* (an adaptor protein). The primed *cheA* will result in autophosphorylation on histidine residue with a corresponding trans-phosphorylation of *cheY* (a primary response regulator) which binds to the flagella motor to initiate chemotaxis movement [1,2]. Other facilitators of chemosensory pathways are *cheR* (methyltransferase), *cheB* (methylesterase), etc. [3]. Often, bacteria perform energy taxis in response to migrating to suitable environments that support their metabolic activities [4], as observed in the rhizosphere of organic manure fertilized maize plants.

Agricultural intensification necessitates the use of chemical fertilizer in boosting soil fertility. This inorganic fertilizer on prolonged application to the soil causes a range of detrimental effects such as eutrophication, greenhouse gas emission, reduction in plants nutrient uptake, and toxicity on soil microbes [5]. The inorganic fertilizer-associated drawbacks prompted soil fertilization with compost, plant/crop residues, animal droppings, etc. These organic substances are not only cost-effective but also are microbiological and environmentally friendly [6,7,8]. The richness of nutrients that could serve as inducers to microbial chemoreceptors causes the improvement in the expression of chemotaxis genes and migration of soil-borne microbes to the nutrient source. Often this microbial migration is towards the rhizosphere. The organic substances given off by the plant roots serve as growth-enhancing nutrients for microbes. The rhizosphere is a spot of high metabolic activities and assembly of a vibrant and unique consortium of microbial communities which actively participate in biogeochemical cycling, plant hormone production, as well as antagonistic antimicrobial chemicals production. Although both beneficial and pathogenic microbes are equally attracted to the rhizosphere, the beneficial ones help to check the activities of the phytopathogens and hence sustain the health of the plants [9,10,11].

Nevertheless, the cascade effects of plant exudates and fertilizer (organic and inorganic) on the priming of chemotaxis genes and attraction of microbial communities to the rhizosphere leads to the promotion and establishment of a disease suppressive soil. This disease suppressive soil operates on a natural principle of the survival of the fittest and competition for dominance. It breeds a stiff microbial competition at the rhizosphere and, depending on the fate of the competitive outcome between the pathogens and the beneficial microbes, determines the chances for development or sustenance of health or death in the plants [12,13]. For instance, the pathogen *Streptomyces* sp. capable of producing thaxtomic substances which aid its infection and initiation of scab disease condition in potato plant is directly controlled by the beneficial bacteria *Pseudomonas fluorescens* LBUM223. This inhibition effect on the *Streptomyces* sp. is due to the antimicrobial substance (Phenazine-1-carboxylic acid) produced by *P. fluorescens* LBUM223, which interferes with the proper cellular function of the pathogen and so results in its inhibition [14]. Antimicrobial substances like 2,4-diacetylphloroglucinol, hydrogen cyanide, chitinase, phenazines, organic acids, as well as iron-chelating substances (siderophores) are responsible for the induction and maintenance of a disease suppressive soil [15,16,17,18]. In this study, we evaluated the functional genes profile abundance involved in chemotaxis and antimicrobial and siderophore producing substances from the maize rhizosphere under organic, inorganic, and untreated control using shotgun metagenomics study. Therefore, we hypothesize that both organic compost at higher dose and low inorganic fertilizer application will not differ from those of the untreated control in the enrichment of chemotaxis genes and antimicrobial agents production genes abundance within the maize rhizosphere. These treatments will enhance the development of disease-suppressive and healthy soil.

## 2. Materials and Methods

### 2.1. Site Description, Samples Collection, and DNA Extraction

The site description and the soil samples were collected as previously described by Enebe and Babalola [19]. The soil chemical constituents are: pH 4.97, phosphorus 10.5 mg/kg, nitrogen 377 mg/kg, potassium 285 mg/kg, calcium 388 mg/kg, magnesium 162 mg/kg, organic carbon 0.36%, and the physical composition was sand 80%, silt 5%, and clay 15%. The farm was divided into plots and treated with inorganic fertilizers (N_20_P_7_K_3_) (120 and 60 kg/ha inorganic fertilizer) and compost manure derived from plant materials, (vegetables and domestic wastes) (8 and 4 tons per hectare). Maize seeds (*Zea mays everta*) were planted in both the fertilized soils and the control. A total of 9 maize plants rhizosphere were sampled (i.e., 3 plants’ rhizosphere samples from each replicate per treatment) (5 treatments x 3 replicate plots per treatment). Sampling was done in early September at the 7th week after germination and the rhizosphere soils were obtained from maize plants at a depth of 0–15 cm from the soil surface using a 5 cm diameter auger. The average temperature was 27.7 °C [20]. At post-sampling, the samples were collected in a sterilized plastic bags and transported to the laboratory for analysis in ice-containing boxes. This was proceeded by the extraction of microbial community DNA from the soil samples using PowerSoil DNA isolation kit (MoBio Laboratories, Incorporation, Carlsbad, CA 92010, USA) following the manufacturer’s guide.

### 2.2. Sequencing of the Community DNA

DNA concentrations in the extracts were measure with Qubit^®^ dsDNA HS Assay Kit (from Life Technologies, Carlsbad, CA, USA). The libraries of the DNA were prepared using Nextera DNA Flex library preparation kit (Illumina Incorporation, San Diego, CA, USA) by following the manufacturer’s user-guide-manual. In each of the samples, 50 nanogram of the DNA was used for the libraries preparation. Adapter sequences were introduced into the samples, and this was followed by fragmentation. The adapter was used during the PCR cycles in the presence of added unique indices. The total libraries’ concentrations generated were quantified using Qubit^®^ dsDNA HS Assay Kit (from Life Technologies, Carlsbad, CA, USA). Additionally, average library’s sizes were evaluated using an analytical machine—Agilent 2100 Bioanalyzer (Agilent Technologies, Santa Clara, CA, USA). Thereafter, the libraries (DNA) was pooled together into an equal-molar ratio of 0.7 nM. The combined DNA were sequenced paired-end for 300 cycles using the sequencing machine—NovaSeq 6000 system (Illumina, San Diego, CA, USA). The sequencing of the DNA was carried out at the Mr DNA molecular research laboratory in USA.

### 2.3. Sequence Processing, Annotation and Statistical Analysis

The metagenomic reads obtained were uploaded to MG-RAST where quality control processes were performed on the reads [21]. The pre-processing of the uploaded reads involved the removal of artificial reads, host-specific sequences, and other ambiguous base pairs. This was proceeded by gene annotation using BLAT algorithm [22] and M5NR database [23]. The annotation of the protein-coding genes were carried out by blasting through M5NR as well as SEED Subsystem level-function. The BlastX was used to perform hit at an E-value cutoff (1 × 10^−5^), minimum alignment length of 15 base pairs, and percentage identity of 60%. The unannotated sequences were not subjected to any further evaluation or analysis. Applied also was the MG-RAST normalization tool to enable us to reduce the possible experimental error. The chemotaxis and disease suppressive genes involved in antibiotics, antifungal, nematicide, and siderophores were manually curated and extracted from the total gene files obtained from the SEED Subsystem. The sequence data were used for statistical analysis, and the chemotaxis, disease suppressive genes’ variances were evaluated using one-way analysis of variance at *p*-value less than 0.05. The abundance and distribution of disease suppressive genes were visualized in a bar chart representation using Microsoft Excel. In addition, the online Circos software was (http://circos.ca/, accessed on 15 February 2020) employed in plotting the chart of chemotaxis genes. Evenness, Simpson, and Shannon diversity indexes were evaluated and contrasted amongst the treatments using a Kruskal–Wallis test. The β diversity was ascertained using principal coordinate analysis (PCoA) based on Euclidean distance-matrix. These analyses were performed using PAST version 3.20 software (Hammer et al. 2001) and CANOCO 5v (Microcomputer Power, Ithaca, NY, USA) for PCoA and principal component analysis (PCA). The sequences are deposited on NCBI SRA dataset, SRA accession: PRJNA607213.

## 3. Results

### 3.1. Metagenomics Dataset

The sequence reads pre-post quality control were 5,558,478 (N2), 9,687,815 (N1), 12,070,719 (Cp4), 7,834,687 (Cn0), 15,575,330 (Cp8), with mean GC percent of 43.11, 64.12, 49.11, 64.11, 63.12% for N2, N1, Cp4, Cn0, and Cp8, respectively. After post quality control process, the sequences that remained are 2,892,203, 8,198,530, 2,945,816, 6,780,803, 13,083,355 sequence reads from N2, N1, Cp4, Cn0, and Cp8 with predicted proteins of known functions comprises of 1,603,127 (61.86%) (Cp4), 5,410,912 (45.25%) (Cp8), 2,834,072 (44.49%) (Cn0), 1,812,036 (69.99%) (N2), 3,418,368 (45.11%) (N1) sequence reads, while 969,988 (37.43%) (Cp4), 6,519,307 (54.52%) (Cp8), 3,523,113 (55.31%) (Cn0), 764,206 (29.52%) (N2), 4,144,168 (54.69%) (N1) were sequences with predicted proteins of unknown functions. The treatments Cp8, N1, and Cn0 enriched the bacterial communities, chemotaxis, and disease suppressive genes at the maize rhizosphere significantly.

### 3.2. Abundant Bacterial Phyla in the Samples

**Figure 1:**
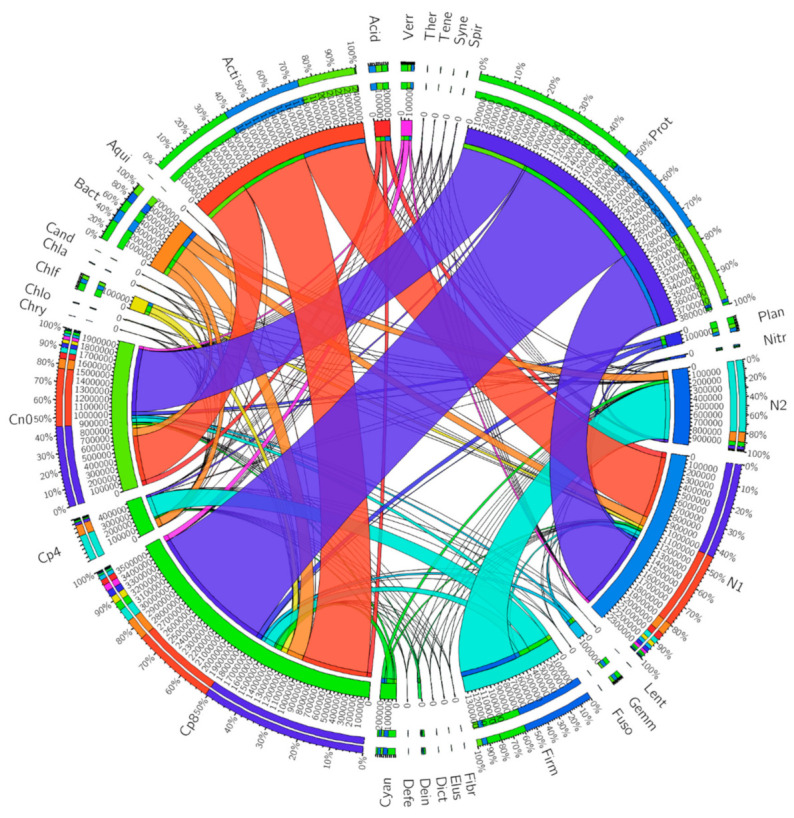
The abundance of bacterial phyla across the treatments from the maize rhizosphere soil samples. Cn0 (control), Cp8 (8 tons/ha compost manure), Cp4 (4 tons/ha compost manure), N2 (120 kg/ha inorganic fertilizer), and N1 (60 kg/ha inorganic fertilizer). The bacteria abbreviations are presented as follows: Acid (*Acidobacteria*), Acti (*Actinobacteria*), Aqui (*Aquificae*), Bact (*Bacteroidetes*), Cand (*Candidatus Poribacteria*), Chla (*Chlamydiae*), Chlo (*Chlorobi*), Chlf (*Chloroflexi*), Chry (*Chrysiogenetes*), Cyan (*Cyanobacteria*), Defe (*Deferribacteres*), Dein (*Deinococcus*-*Thermus*), Dict (*Dictyoglomi*), Elus (*Elusimicrobia*), Fibr (*Fibrobacteres*), Firm (*Firmicutes*), Fuso (*Fusobacteria*), Gemm (*Gemmatimonadetes*), Lent (*Lentisphaerae*), Nitr (*Nitrospirae*), Plan (*Planctomycetes*), Prot (*Proteobacteria*), Spir (*Spirochaetes*), Syne (*Synergistetes*), Tene (*Tenericutes*), Ther (*Thermotogae*), Verr (*Verrucomicrobia*). Adapted from Enebe and Babalola [24].

The microbial sequenced genomes present at the maize rhizosphere have an abundant of Proteobacteria and Actinobacteria which was dominant in Cp8, Cn0, and N1 samples. Firmicutes, on the other hand, are the most abundant bacterial phyla in N2 and Cp4 samples. There was nearly equal distribution in proportion of Bacteriodetes in all the samples but Cp8 sample had the highest recorded abundance (Figure 1).

### 3.3. Chemotaxis Genes Present at the Maize Rhizosphere

**Figure 2:**
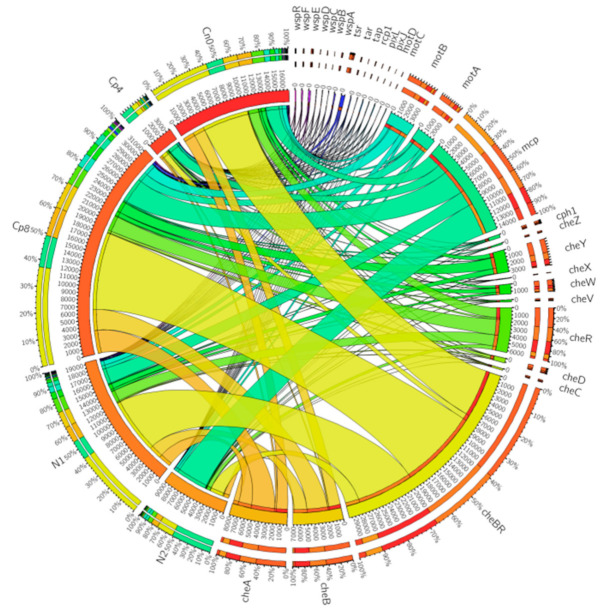
The distribution of chemotaxis genes in maize rhizosphere soil samples under fertilization and control conditions. Cn0 (control), Cp8 (8 tons/ha compost manure), Cp4 (4 tons/ha compost manure), N2 (120 kg/ha inorganic fertilizer), and N1 (60 kg/ha inorganic fertilizer). The gene symbols are depicted as follows: (*cheBR*) two-component system, chemotaxis family, CheB/CheR fusion protein, (*mcp*) methyl-accepting chemotaxis protein, (*cheA*) two-component system, chemotaxis family, sensor kinase CheA, (*cheB*) two-component system, chemotaxis family, response regulator CheB, (*cheR*) chemotaxis protein methyltransferase CheR, (*motB*) chemotaxis protein MotB, (*cheY*) two-component system, chemotaxis family, response regulator CheY, (*motA*) chemotaxis protein MotA, (*cheW*) purine-binding chemotaxis protein CheW, (*cheD*) chemotaxis protein CheD, (*tsr*) methyl-accepting chemotaxis protein I, serine sensor receptor, (*cph1*) two-component system, chemotaxis family, sensor kinase Cph1, (*cheV*) two-component system, chemotaxis family, response regulator CheV, (*cheZ*) chemotaxis protein CheZ, (*cheC*) chemotaxis protein CheC, (*wspE*) two-component system, chemotaxis family, sensor histidine kinase and response regulator WspE, (*tar*) methyl-accepting chemotaxis protein II, aspartate sensor receptor, (*wspR*) two-component system, chemotaxis family, response regulator WspR, (*wspF*) two-component system, chemotaxis family, response regulator WspF, (*cheX*) chemotaxis protein CheX, (*rcp1*) two-component system, chemotaxis family, response regulator Rcp1, (*wspA*) methyl-accepting chemotaxis protein WspA, (*motC*) chemotaxis protein MotC, (*wspC*) chemotaxis protein methyltransferase WspC, (*pixJ*) methyl-accepting chemotaxis protein PixJ, (*wspD*) chemotaxis-related protein WspD, (*pixL*) two-component system, chemotaxis family, sensor histidine kinase and response regulator PixL, (*motD*) chemotaxis protein MotD, (*tap*) methyl-accepting chemotaxis protein IV, peptide sensor receptor, (*wspB*) chemotaxis-related protein WspB.

The metagenomics sequences from the maize rhizosphere soil samples under the treatments containing the chemotaxis genes differ significantly (*p* < 0.05) amongst the treatments Cp8, N1, and Cn0 from treatments N2 and Cp4 (Figure 2). The abundance of these genes also varies among the treatments. Of the chemotaxis genes identified, *cheBR* fusion proteins involved in signal transduction of the two-component systems, followed by *mcp* (methyl accepting chemotaxis protein), *cheA* (Histidine kinase), *cheB* (methyl esterase), *cheR* (methyl transferase), *mot* B and A (chemotaxis proteins *motB* and *motA*), *cheY* (response regulator), as well as *cheW* (coupling protein) were the most abundant chemotaxis genes present in the sequenced samples.

### 3.4. Antimicrobial and Siderophore Genes Contributing to Disease Suppressive Soil

**Figure 3:**
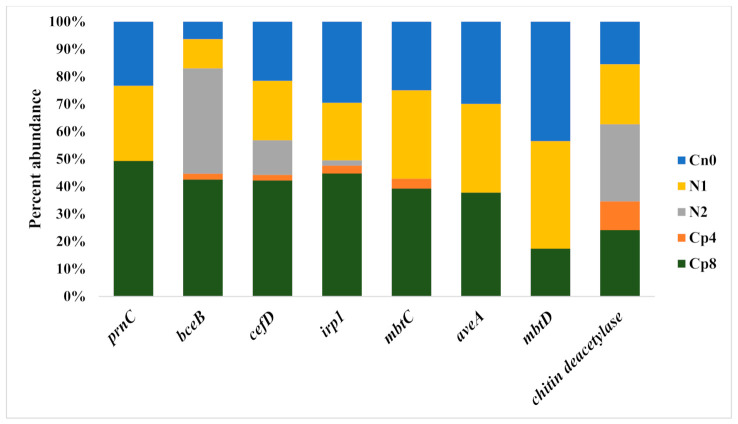
The percentage distribution of antimicrobial and siderophores producing genes within the rhizosphere soil of maize treated with organic, inorganic fertilizers and control treatments Cn0 (control), Cp8 (8 tons/ha compost manure), Cp4 (4 tons/ha compost manure), N2 (120 kg/ha inorganic fertilizer) and N1 (60 kg/ha inorganic fertilizer). prnC (FADH2 O2-dependent halogenase II), bceB (bacitracin transport system permease protein), cefD (isopenicillin-N epimerase), irp1 (yersiniabactin nonribosomal peptide/polyketides), mbtC (mycobactin polyketide synthase MbtC), aveA (type 1 polyketide synthase AVES), mbtD (mycobactin polyketide synthase MbtD).

Under treatments and untreated fertilization, the metagenomics sequences containing antimicrobial—siderophore genes implicated in the development of a disease suppressive soil are *prnC* (FADH2 O2—dependent halogenase II), *bceB* (bacitracin transport system permease protein), *cefD* (isopenicillin N epimerase), *irp1* (yersiniabactin nonribosomal protein), *mbtC* (mycobactin polyketide synthetase), *aveA* (type 1 polyketide synthase AVES), *mbtD* (mycobactin polyketide synthase) and chitin deacetylase. They are very abundant in the soil at varying concentrations. *bceB*, *cefD*, and *irp1* did not differ in Cp8, and Cp4, but the rest of the genes differed across the treatments (Figure 3).

### 3.5. Diversity (α and β) Estimation of the Chemotaxis and the Disease Suppressive Genes

**Figure 4:**
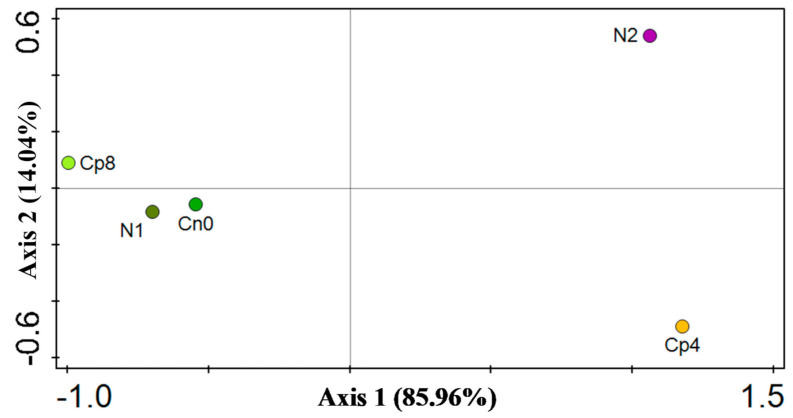
PCoA analysis showing the β diversity of chemotaxis genes within the fertilized and unfertilized maize rhizosphere soil samples Cn0 (control), Cp8 (8 tons/ha compost manure), Cp4 (4 tons/ha compost manure), N2 (120 kg/ha inorganic fertilizer), and N1 (60 kg/ha inorganic fertilizer).

**Figure 5:**
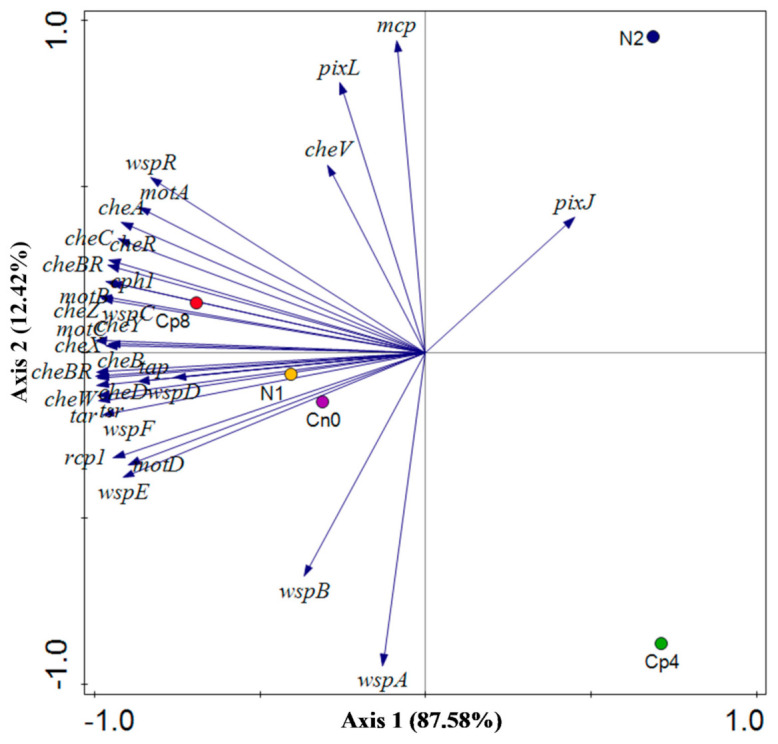
PCA—principal component analysis of the chemotaxis genes abundance within the maize rhizosphere treated with organic, inorganic fertilizer, and untreated control Cn0 (control), Cp8 (8 tons/ha compost manure), Cp4 (4 tons/ha compost manure), N2 (120 kg/ha inorganic fertilizer) and N1 (60 kg/ha inorganic fertilizer).

**Figure 6:**
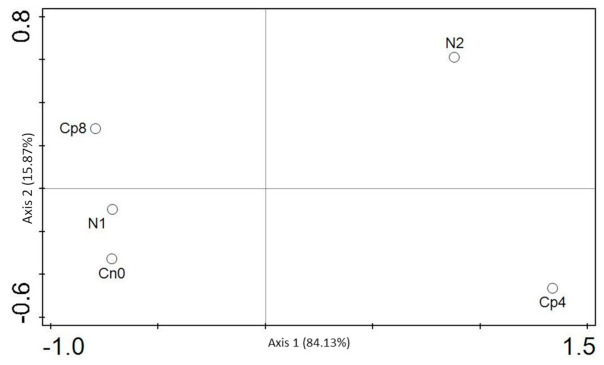
Principal component analysis for the identified disease suppressive genes in the maize rhizosphere treated with organic and inorganic fertilizers. Cn0 (control), Cp8 (8 tons/ha compost manure), Cp4 (4 tons/ha compost manure), N2 (120 kg/ha inorganic fertilizer), and N1 (60 kg/ha inorganic fertilizer).

**Figure 7:**
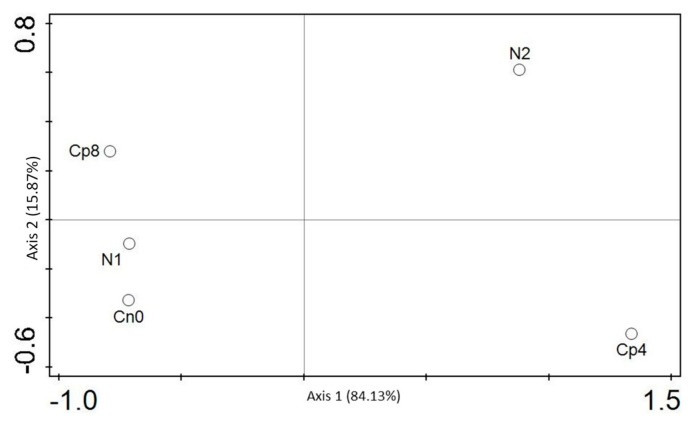
Principal coordinate analysis for the disease suppressive genes present at the maize rhizosphere under fertilization treatments. Cn0 (control), Cp8 (8 tons/ha compost manure), Cp4 (4 tons/ha compost manure), N2 (120 kg/ha inorganic fertilizer), and N1 (60 kg/ha inorganic fertilizer).

The diversity indices were depicted by Shannon, Simpson, and evenness for α diversity of the chemotaxis genes. Shannon, Simpson, and evenness diversity indexes clearly showed that there were significant differences (*p* < 0.001) for the chemotaxis functional genes α diversity. Moreover, there was no difference in the β diversity, which is, the diversity between the unfertilized and fertilized (Cp8, Cp4, and N1) rhizosphere soils (Table 1) and depicted by the principal coordinate analysis—PCoA (Figure 4). Additionally, the α diversity indices based on Kruskal–Wallis test for disease suppressive genes differed significantly (*p* < 0.05). In addition, the principal component analysis (PCA) was performed to show the distribution of chemotaxis genes between the treatments and the control samples (Figure 5). PCA and PCoA were performed for disease suppressive genes (Figure 6 and Figure 7) and also for bacterial phyla (Figures S1 and S2).

## 4. Discussion

Soil fertility is a function of the level of organic substances present in the soil, and it influences the activities of soil-borne microbes, plant growth performance, and a host of other beneficial ecological functions [25,26]. The microbes present in the rhizosphere of plants have been implicated in plant nutrient uptake, growth hormones production, scavenging for parasitic pathogens, competing for nutrients with pathogens, induction of systemic, and acquired resistance by the plants to pathogens, enabling plants to tolerate abiotic stress, and perform biogeochemical cycling processes [12,27,28,29]. The bacteria belonging to the phyla *Firmicutes* and *Chlamydiae* are the major driver separating N2 and Cp4 from the control and other treatments. At the same time, *Cyanobacteria* and *Bacteroidetes* are responsible for the observed separation between N2 and Cp4. The factors, along with the negative values of axis 1 separated Cp8 from N1 and Cn0. Additionally, analysis of the bacterial phyla shown in PCA plot has a strong positive loading for *Firmicutes* and *Chlamydia* and negative loading for *Fusobacteria, Aquificae, Chlorobi, Proteobacteria, Nitrospirae, Actinobacteria*, and *Acidobacteria*. This reflects an increased abundance of plant growth-promoting bacteria at the rhizosphere of maize plants at both positive and negative axis 1. For instance, *Proteobacteria, Actinobacteria*, and *Firmicutes* are excellent chitinase-producing bacteria in the soil [30]. Axis 2 shows strong positive loadings for *Cyanobacteria* and *Bacteroidetes* at positive axis 2. These microbes are good organic matter degraders (performed by *Bacteroidetes*) [31] and fix carbon and nitrogen in the soil (*Cyanobacteria*) [32].

The bacterial species belonging to the phyla Proteobacteria have been found to participate in soil functions such as nutrient cycling and soil health maintenance. They are gram-negative bacteria that are most abundant in agricultural soils, as reported by other studies [33,34,35]. The presence of this group of bacteria in Cp8, N1, and Cn0 samples is consistent with the findings by Fierer et al. [36] and Mhete et al. [37]. Actinobacteria, on the other hand, are gram-positive bacteria that involves in the biogeochemical cycling of nutrients and production of various secondary metabolites [38]. Bacteroidetes, also are very abundant in the soil and anaerobic environments. They inhabit the plants’ rhizosphere and participate in the maintenance of soil health, degradation of organic polymers, and in nutrients cycling [36,39] and their population increase with an increase in soil nutrients or fertilization [40]. In addition, Firmicutes have been reported to be very abundant in slightly acidic soil [41], as also found in this work.

In this metagenomics study, however, we evaluated the response of maize microbial functional gene diversity involved in chemotaxis, antibiotics, siderophores, and antifungals production within the rhizosphere of maize plants under inorganic compost fertilizer and unfertilized conditions. The results clearly show that fertilization treatments at higher compost manure and lower inorganic fertilizer doses, as well as maize plants in the unfertilized soil through rhizosphere effects, exert varying influences on the abundance of chemotaxis siderophores, antifungal, and antibiotics synthesizing genes present in the samples.

Maize rhizosphere soil treated with compost at a concentration of 8 tons per hectare showed the highest chemotaxis, antibiotics, siderophores, and antifungal gene richness, followed by the lower inorganic fertilizer and the control. It is not yet clearly understood the extent to which the compost manure contributes to the soil enrichment of the chemotaxis and disease suppressive genes in the soil, making it difficult to arrive at a definite conclusion when comparing organic and inorganic fertilizer influence on the bacterial motility and disease suppression in the soil. However, organic manure has been shown to stimulate the antagonistic levels of the bacterial community in the soil, which in turn enhances disease suppression of soil-borne microbial pathogens [42,43,44]. Additionally, there exists an inversely proportional relation between inorganic fertilizer application and the diversity, as well as the richness of microbes. This explains the rationale behind the observed abundance of these genes at the rhizosphere treated with lower inorganic fertilizer [45]. However, the genes involved in chemotaxis and disease suppression were also enriched by the maize plants. It could be assumed that through rhizodeposition, the plants were able to attract a consortium of specialized microbes [46] or through the production of signaling chemical compounds such as strigolactones capable of attracting arbuscular mycorrhizal fungi [47] or *Pseudomonas* attractant compound such as benzoxazinoids [48] and a host of other chemicals that boost the richness and diversity of the rhizosphere microbial population and their corresponding chemotactic and disease suppressive genes.

The observed decrease in the abundance of the chemotaxis genes at a higher dose of inorganic fertilizer could be explained by the indirect soil pH modification effects [49] and the roots’ functional and structural adjustment in response to the fertilizer’s acidification effects that may result in a change of the rhizosphere microbial population, activities, and their colonization rate [50].

However, the *pixJ* is the primary driver to separate the N2 and Cp4 from the other control and treated samples. While *mcp, pixL, cheV, wspB, wspA* are the forces that separate N2 and Cp4. The first three work oppositely from the last two. The factors along the negative values of Axis 1 distinguished the Cp8 from N1 and Cn0.

Therefore, axis 1 of the principal component analysis of the chemotaxis genes shows a strong positive loading for methyl-accepting chemotaxis protein (*pixJ*) and a strong negative loading for *motA, motB, cheBR, cheW, tar*, etc. This analysis of bacterial chemotaxis genes reflects relatively strong sensing of environmental and intracellular signals by the bacteria in response to nutrients gradients in the soil at positive axis 1 [51], and increasing signaling transmission cascade, excitation, phosphorylation, dephosphorylation, deamination, and methylation (that is, signal detection, activation of kinase, phosphate group transfer, and generation of response) [52] that facilitate bacteria migration from the bulk soil to the rhizosphere at negative axis 1. In axis 2, there are strong positive loadings for methyl-accepting chemotaxis protein (*mcp*), two-component, chemotaxis family, sensor histidine kinase and response regulator (*pixL*), and two-component system, chemotaxis family, response regulator (*cheV*). This reflects signal sensing from the environment and transmission of those signals through phosphate group transfer from histidine (of histidine kinase enzyme) to aspartate component of the response regulator. They participate in cellular and environmental signal detection and priming of bacterial cellular response such as flagella rotation and bacterial motility [53,54]. Methyl-accepting chemotaxis protein (*wspA*) and chemotaxis-related protein (*wspB*) have strong negative loadings at axis 2. This implies an increase in signal sensing and signal transmission to the effector sites within the bacterial cells [55]. Our result has shown that nutrients gradients are the strongest determining factor in the abundance of bacterial chemotaxis genes under organic fertilization but with opposite effects under inorganic fertilization. Treatment of soil with 8 tons per hectare of compost manure increase the nutrients gradient and chemical signals that prompted bacterial signal detection, response, and motility towards the nutrient rich rhizosphere. Small quantity of inorganic fertilizer and the control (i.e., maize rhizosphere without any fertilization) equally enhanced the expression of these genes.

Our results also suggest that the chemotaxis genes *mcp* (methyl-accepting chemotaxis protein), which is a chemoreceptor and *cheBR* (cheB/cheR fusion protein), *cheA* (histidine kinase), *cheB* (methyl esterase), *cheR* (methyl transferase), *motB* and A (chemotaxis protein *motB*, and *MotA*), *cheY* (response regulator), *cheW* (coupling protein) are cytoplasmic proteins [3,56,57,58] which were the most abundant in the high compost, lower inorganic fertilizer, and the control. They facilitate the motility of the microbes within the soil rhizosphere environment. It is known that the process of chemotaxis involved in the flagellar rotation is adenosine triphosphate (ATP) and electron transport dependent [59], which explains why a higher dose of compost with abundant nutrient supply efficiency could influence the chemotaxis activities of the rhizosphere microbes above other treatments. However, the maize plants, as well as the lower dose of inorganic fertilizers, also enriched these chemotaxis genes. Their abundance and expression could be proportional to the presence of ligand molecules capable of binding to the chemoreceptors, which are as follows: phosphate ions, phytohormones, sugars, amino acids, oxygen molecules, hydrocarbon molecules, quorum sensing signaling molecules, etc. [60,61,62,63,64]. Although these molecules could attract both pathogenic and beneficial microbes, the observed disease suppressive genes present in the treatments have shown that compost manure (Cp8), N1 (60 Kg/ha inorganic fertilizer), and maize plant (Cn0) are capable of attracting disease suppressive microbes which could facilitate the development of a disease suppressive soil.

The gene *cefD* is the key driving factor that separates Cp8, N1, and Cn0 from N2 and Cp4 samples. Chitin deacetylase and *bceB* are the major drivers that separate N2 and Cp4. Axis 1 has a strong negative loading for *prnC, aveA, irp1, mbtC*, and *mbtD* genes. This depicts an increase in antifungal (pyrrolnitrin), nematicides (avermectin), and siderophores (carboxymycobactins and yersiniabactin) [65,66] in the maize rhizosphere at negative axis 1. At positive axis 2, there is a strong positive loading for *bceB* and chitin deacetylase. This reflect an abundance of antibiotics and chitin degrading enzymes in the maize rhizosphere. These antibiotics, antifungal, nematicides, and siderophores producing genes are supported by soil fertilization with a high quantity of compost manure, and it favors plants protection from soil pathogens invasion and enhances soil health.

The observed siderophores genes present in the rhizosphere soil treated with the compost and inorganic fertilizer and the control are *mbtC*, *mbtD* (mycobactin polyketide synthases), and *irp1* (yersiniabactin nonribosomal protein). Siderophores are chemical compounds with a high affinity for iron molecules that are used by microbes to scavenge iron and create environments of iron deficiency. This iron-deficient environment impairs microbial DNA synthesis and respiration, requiring iron molecules to function effectively [65,67]. Alongside siderophore genes abundance, antibiotics and antifungal genes (*prnC, cefD, aveA*) were abundant in the samples. *aveA* genes are responsible for producing avermectin (a nematicide) capable of inhibiting nematodes [68]. *prnC* (FADH2-dependent halogenase II) is responsible for antifungal pyrrolnitrin production [69]. *cefD* (isopenicillin N epimerase) is involved in penicillin production [70]. This study clearly demonstrated the influence of compost manure at higher treatment, low inorganic fertilizer and maize plants without any fertilization on the microbial chemotaxis genes abundance and disease suppressive genes. Therefore, understanding the effects of fertilizer and maize plants on the enrichment of chemotaxis genes and functional genes involved in disease suppression will be useful in the actualization of sustainable agriculture through the manipulation of the soil microbiomes.

## 5. Conclusions

This study clearly shows the effects of fertilization on the abundance of chemotaxis and disease suppressive genes in the maize rhizosphere. Our results revealed that treating agricultural soil with higher doses of compost manure derived from plant materials and domestic waste or lower inorganic fertilizer separately will lead to the enrichment of the rhizosphere with chemotaxis, antibiotics, siderophores, antifungal, and nematicides synthesizing genes. Maize plants, on the other hand, have been proven to exert significant rhizosphere effects in attracting and enriching the rhizosphere with beneficial functional microbial genes, as well as microbes. Therefore, to achieve healthy soil, we recommend fertilizing the soil with either compost manure at 8 tons per hectare or with a low quantity of inorganic fertilizer (60 kg/ha). They enhance the development of a disease suppressive soil. This notwithstanding, intercropping of maize plants with a disease susceptible crop in a disease conducive soil could be a good alternative in attracting beneficial microbes to combat the invasiveness of the pathogens and hence the achievement of sustainable agriculture.

## Figures and Tables

**Table 1:** Diversity indexes of chemotaxis genes within the fertilized and unfertilized soils from maize rhizosphere Cn0 (control), Cp8 (8 tons/ha compost manure), Cp4 (4 tons/ha compost manure), N2 (120 kg/ha inorganic fertilizer), and N1 (60 kg/ha inorganic fertilizer)

	Cp8	Cp4	N2	N1	Cn0	*p* Value
Simpson	0.8377	0.8025	0.6948	0.8065	0.8139	5.7 × 10^−4^
Shannon	2.219	2.068	1.649	2.084	2.105	
Evenness	0.2967	0.2824	0.1928	0.2593	0.2648	

## Data Availability

The data is deposited at NCBI SRA under the accession number: PRJNA607213.

## Supplementary Materials

The following are available online at https://www.mdpi.com/article/10.3390/genes12040535/s1, Figure S1: PCoA (Principal Coordinate Analysis) showing the beta diversity between the maize rhizosphere soils at different fertilization regimes, Cn0 (control), N60 (60 kg/ha NPK), N120 (120 kg/ha NPK), Cp8 (8 tons/ha compost), and Cp4 (4 tons/ha compost manure). Adapted from Enebe and Babalola [24] with slight modification. Figure S2: PCA (principal component analysis) of the bacterial community structure associated with maize rhizosphere grown under different fertilization and unfertilized regimes (compost 8 tons/ha—Cp8, compost 4 tons/ha—Cp4, control—Cn0, 60 kg/ha inorganic fertilizer—N60, 120 kg/ha inorganic fertilizer—N120), showing treatments as the key factors influencing the structural shift and shape of bacterial community at the rhizosphere soil samples. The percentages represent the observed variations, and the bacterial abbreviations are Acid (*Acidobacteria*), Acti (*Actinobacteria*), Aqui (*Aquificae*), Bact (*Bacteroidetes*), Cyan (*Cyanobacteria*), Chla (*Chlamydiae*), Chlo (*Chlorobi*), Firm (*Firmicutes*), Fuso (*Fusobacteria*), Nitr (*Nitrospirae*), Prot (*Proteobacteria*). Adapted from Enebe and Babalola [24] with slight modification.

## References

[1] Parkinson J.S., Hazelbauer G.L., Falke J.J. (2015). Signaling and sensory adaptation in Escherichia coli chemoreceptors: 2015 update. Trends Microbiol..

[2] Sourjik V., Wingreen N.S. (2012). Responding to chemical gradients: Bacterial chemotaxis. Curr. Opin. Cell Biol..

[3] Wuichet K., Zhulin I.B. (2010). Origins and diversification of a complex signal transduction system in prokaryotes. Sci. Signal..

[4] Schweinitzer T., Josenhans C. (2010). Bacterial energy taxis: A global strategy?. Arch. Microbiol..

[5] Zhu Y.-G., Su J.-Q., Cao Z., Xue K., Quensen J., Guo G.-X., Yang Y.-F., Zhou J., Chu H.-Y., Tiedje J.M. (2016). A buried Neolithic paddy soil reveals loss of microbial functional diversity after modern rice cultivation. Sci. Bull..

[6] Tejada M., Gonzalez J., García-Martínez A., Parrado J. (2008). Effects of different green manures on soil biological properties and maize yield. Bioresour. Technol..

[7] Bhattacharyya R., Kundu S., Prakash V., Gupta H. (2008). Sustainability under combined application of mineral and organic fertilizers in a rainfed soybean–wheat system of the Indian Himalayas. Eur. J. Agron..

[8] Ajilogba C.F., Babalola O.O., Ahmad F. (2013). Antagonistic effects of Bacillus species in biocontrol of tomato Fusarium wilt. Stud. Ethno Med..

[9] Mendes R., Garbeva P., Raaijmakers J.M. (2013). The rhizosphere microbiome: Significance of plant beneficial, plant pathogenic, and human pathogenic microorganisms. FEMS Microbiol. Rev..

[10] Marschner P., Crowley D., Yang C.H. (2004). Development of specific rhizosphere bacterial communities in relation to plant species, nutrition and soil type. Plant Soil.

[11] Babalola O.O., Glick B.R. (2012). The use of microbial inoculants in African agriculture: Current practice and future prospects. J. Food Agric. Environ..

[12] Enebe M.C., Babalola O.O. (2019). The impact of microbes in the orchestration of plants’ resistance to biotic stress: A disease management approach. Appl. Microbiol. Biotechnol..

[13] Adegboye M.F., Babalola O.O. (2015). Evaluation of antibiotic biosynthetic potential of actinomycete isolates to produce antimicrobial agents. Microbiol. Res. J. Int..

[14] Arseneault T., Goyer C., Filion M. (2015). Pseudomonas fluorescens LBUM223 increases potato yield and reduces common scab symptoms in the field. Phytopathology.

[15] Latz E., Eisenhauer N., Rall B.C., Allan E., Roscher C., Scheu S., Jousset A. (2012). Plant diversity improves protection against soil-borne pathogens by fostering antagonistic bacterial communities. J. Ecol..

[16] Raaijmakers J.M., Mazzola M. (2012). Diversity and natural functions of antibiotics produced by beneficial and plant pathogenic bacteria. Annu. Rev. Phytopathol..

[17] Ajayi A., Onibokun E., George F., Atolagbe O. (2016). Isolation and characterization of chitinolytic bacteria for chitinase production from the African Catfish, Clarias gariepinus (Burchell, 1822). Res. J. Microbiol..

[18] Adegboye M.F., Babalola O.O. (2012). Taxonomy and ecology of antibiotic producing actinomycetes. Afr. J. Agric. Res..

[19] Enebe M.C., Babalola O.O. (2021). Soil fertilization affects the abundance and distribution of carbon and nitrogen cycling genes in the maize rhizosphere. AMB Express.

[20] Adebayo A.R., Kutu F.R., Sebetha E.T. (2020). Data on root system architecture of water efficient maize as affected by different nitrogen fertilizer rates and plant density. Data Brief.

[21] Meyer F., Paarmann D., D’Souza M., Olson R., Glass E.M., Kubal M., Paczian T., Rodriguez A., Stevens R., Wilke A. (2008). The metagenomics RAST server—A public resource for the automatic phylogenetic and functional analysis of metagenomes. BMC Bioinform..

[22] Kent W.J. (2002). BLAT—the BLAST-like alignment tool. Genome Res..

[23] Wilke A., Harrison T., Wilkening J., Field D., Glass E.M., Kyrpides N., Mavrommatis K., Meyer F. (2012). The M5nr: A novel non-redundant database containing protein sequences and annotations from multiple sources and associated tools. BMC Bioinform..

[24] Enebe M.C., Babalola O.O. (2020). Effects of inorganic and organic treatments on the microbial community of maize rhizosphere by a shotgun metagenomics approach. Ann. Microbiol..

[25] Marschner P., Kandeler E., Marschner B. (2003). Structure and function of the soil microbial community in a long-term fertilizer experiment. Soil Biol. Biochem..

[26] Lee S.B., Lee C.H., Jung K.Y., Do Park K., Lee D., Kim P.J. (2009). Changes of soil organic carbon and its fractions in relation to soil physical properties in a long-term fertilized paddy. Soil Tillage Res..

[27] Enebe M.C., Babalola O.O. (2018). The influence of plant growth-promoting rhizobacteria in plant tolerance to abiotic stress: A survival strategy. Appl. Microbiol. Biotechnol..

[28] Pieterse C.M., Zamioudis C., Berendsen R.L., Weller D.M., Van Wees S.C., Bakker P.A. (2014). Induced systemic resistance by beneficial microbes. Annu. Rev. Phytopathol..

[29] Berendsen R.L., Pieterse C.M., Bakker P.A. (2012). The rhizosphere microbiome and plant health. Trends Plant Sci..

[30] Beier S., Bertilsson S. (2013). Bacterial chitin degradation—mechanisms and ecophysiological strategies. Front. Microbiol..

[31] Thomas F., Hehemann J.-H., Rebuffet E., Czjzek M., Michel G. (2011). Environmental and gut bacteroidetes: The food connection. Front. Microbiol..

[32] Chamizo S., Mugnai G., Rossi F., Certini G., De Philippis R. (2018). Cyanobacteria inoculation improves soil stability and fertility on different textured soils: Gaining insights for applicability in soil restoration. Front. Environ. Sci..

[33] Borneman J., Skroch P.W., O’Sullivan K.M., Palus J.A., Rumjanek N.G., Jansen J.L., Nienhuis J., Triplett E.W. (1996). Molecular microbial diversity of an agricultural soil in Wisconsin. Appl. Environ. Microbiol..

[34] Smit E., Leeflang P., Gommans S., van den Broek J., van Mil S., Wernars K. (2001). Diversity and seasonal fluctuations of the dominant members of the bacterial soil community in a wheat field as determined by cultivation and molecular methods. Appl. Environ. Microbiol..

[35] Valinsky L., Della Vedova G., Scupham A.J., Alvey S., Figueroa A., Yin B., Hartin R.J., Chrobak M., Crowley D.E., Jiang T. (2002). Analysis of bacterial community composition by oligonucleotide fingerprinting of rRNA genes. Appl. Environ. Microbiol..

[36] Fierer N., Bradford M.A., Jackson R.B. (2007). Toward an ecological classification of soil bacteria. Ecology.

[37] Mhete M., Eze P.N., Rahube T.O., Akinyemi F.O. (2020). Soil properties influence bacterial abundance and diversity under different land-use regimes in semi-arid environments. Sci. Afr..

[38] Shivlata L., Tulasi S. (2015). Thermophilic and alkaliphilic Actinobacteria: Biology and potential applications. Front. Microbiol..

[39] Martínez-Alonso M., Escolano Sánchez J., Montesinos Seguí E., Gaju N. (2010). Diversity of the bacterial community in the surface soil of a pear orchard based on 16S rRNA gene analysis. Int. Microbiol..

[40] Nemergut D.R., Townsend A.R., Sattin S.R., Freeman K.R., Fierer N., Neff J.C., Bowman W.D., Schadt C.W., Weintraub M.N., Schmidt S.K. (2008). The effects of chronic nitrogen fertilization on alpine tundra soil microbial communities: Implications for carbon and nitrogen cycling. Environ. Microbiol..

[41] Kuramae E.E., Gamper H.A., Yergeau E., Piceno Y.M., Brodie E.L., DeSantis T.Z., Andersen G.L., Van Veen J.A., Kowalchuk G.A. (2010). Microbial secondary succession in a chronosequence of chalk grasslands. ISME J..

[42] Perez C., Dill-Macky R., Kinkel L.L. (2008). Management of soil microbial communities to enhance populations of Fusarium graminearum-antagonists in soil. Plant Soil.

[43] Huang J., Li H., Yuan H. (2006). Effect of organic amendments on Verticillium wilt of cotton. Crop. Prot..

[44] Ahmad F., Babalola O.O., Siddiqui M.A. (2012). Integrated Approach for Management of Nematodes in Chickpea. J. Pure Appl. Microbiol..

[45] Zeng J., Liu X., Song L., Lin X., Zhang H., Shen C., Chu H. (2016). Nitrogen fertilization directly affects soil bacterial diversity and indirectly affects bacterial community composition. Soil Biol. Biochem..

[46] Philippot L., Raaijmakers J.M., Lemanceau P., Van Der Putten W.H. (2013). Going back to the roots: The microbial ecology of the rhizosphere. Nat. Rev. Microbiol..

[47] Bouwmeester H.J., Roux C., Lopez-Raez J.A., Becard G. (2007). Rhizosphere communication of plants, parasitic plants and AM fungi. Trends Plant Sci..

[48] Neal A.L., Ahmad S., Gordon-Weeks R., Ton J. (2012). Benzoxazinoids in root exudates of maize attract Pseudomonas putida to the rhizosphere. PLoS ONE.

[49] Zhang Y., Shen H., He X., Thomas B.W., Lupwayi N.Z., Hao X., Thomas M.C., Shi X. (2017). Fertilization shapes bacterial community structure by alteration of soil pH. Front. Microbiol..

[50] Geisseler D., Scow K.M. (2014). Long-term effects of mineral fertilizers on soil microorganisms—A review. Soil Biol. Biochem..

[51] Zhulin I.B. (2001). The Superfamily of Chemotaxis Transducers: From Physiology to Genomics and Back. Sci. Direct.

[52] Chao X., Muff T.J., Park S.-Y., Zhang S., Pollard A.M., Ordal G.W., Bilwes A.M., Crane B.R. (2006). A receptor-modifying deamidase in complex with a signaling phosphatase reveals reciprocal regulation. Cell.

[53] Igo M.M., Ninfa A.J., Stock J.B., Silhavy T.J. (1989). Phosphorylation and dephosphorylation of a bacterial transcriptional activator by a transmembrane receptor. Genes Dev..

[54] Zschiedrich C.P., Keidel V., Szurmant H. (2016). Molecular mechanisms of two-component signal transduction. J. Mol. Biol..

[55] Güvener Z.T., Harwood C.S. (2007). Subcellular location characteristics of the Pseudomonas aeruginosa GGDEF protein, WspR, indicate that it produces cyclic-di-GMP in response to growth on surfaces. Mol. Microbiol..

[56] Sourjik V. (2004). Receptor clustering and signal processing in E. coli chemotaxis. Trends Microbiol..

[57] Porter S.L., Wadhams G.H., Armitage J.P. (2011). Signal processing in complex chemotaxis pathways. Nat. Rev. Microbiol..

[58] Stader J., Matsumura P., Vacante D., Dean G., Macnab R. (1986). Nucleotide sequence of the Escherichia coli motB gene and site-limited incorporation of its product into the cytoplasmic membrane. J. Bacteriol..

[59] Zhao X., Norris S.J., Liu J. (2014). Molecular architecture of the bacterial flagellar motor in cells. Biochemistry.

[60] Matilla M.A., Krell T. (2017). Chemoreceptor-based signal sensing. Curr. Opin. Biotechnol..

[61] Antunez-Lamas M., Cabrera E., Lopez-Solanilla E., Solano R., González-Melendi P., Chico J.M., Toth I., Birch P., Pritchard L., Liu H. (2009). Bacterial chemoattraction towards jasmonate plays a role in the entry of Dickeya dadantii through wounded tissues. Mol. Microbiol..

[62] Hegde M., Englert D.L., Schrock S., Cohn W.B., Vogt C., Wood T.K., Manson M.D., Jayaraman A. (2011). Chemotaxis to the quorum-sensing signal AI-2 requires the Tsr chemoreceptor and the periplasmic LsrB AI-2-binding protein. J. Bacteriol..

[63] Wu H., Kato J., Kuroda A., Ikeda T., Takiguchi N., Ohtake H. (2000). Identification and characterization of two chemotactic transducers for inorganic phosphate in Pseudomonas aeruginosa. J. Bacteriol..

[64] Esuola C.O., Babalola O.O., Heine T., Schwabe R., Schlömann M., Tischler D. (2016). Identification and characterization of a FAD-dependent putrescine N-hydroxylase (GorA) from Gordonia rubripertincta CWB2. J. Mol. Catal. B Enzym..

[65] Ahmadi M.K., Fawaz S., Jones C.H., Zhang G., Pfeifer B.A. (2015). Total biosynthesis and diverse applications of the nonribosomal peptide-polyketide siderophore yersiniabactin. Appl. Environ. Microbiol..

[66] Sritharan M. (2016). Iron homeostasis in Mycobacterium tuberculosis: Mechanistic insights into siderophore-mediated iron uptake. J. Bacteriol..

[67] Perry R.D., Balbo P.B., Jones H.A., Fetherston J.D., DeMoll E. (1999). Yersiniabactin from Yersinia pestis: Biochemical characterization of the siderophore and its role in iron transport and regulation. Microbiology.

[68] Ikeda H., Nonomiya T., Usami M., Ohta T., Ōmura S. (1999). Organization of the biosynthetic gene cluster for the polyketide anthelmintic macrolide avermectin in Streptomyces avermitilis. Proc. Natl. Acad. Sci. USA.

[69] Van Pée K.-H., Zehner S. (2003). Enzymology and Molecular Genetics of Biological Halogenation. Natural Production of Organohalogen Compounds.

[70] Kovacevic S., Tobin M.B., Miller J.R. (1990). The beta-lactam biosynthesis genes for isopenicillin N epimerase and deacetoxycephalosporin C synthetase are expressed from a single transcript in Streptomyces clavuligerus. J. Bacteriol..

