# Supplementary material for: Metagenomics Assessment of Soil Fertilization on the Chemotaxis and Disease Suppressive Genes Abundance in the Maize Rhizosphere
Source: Genes (Basel). 2021 Apr 7;12(4):535. doi: 10.3390/genes12040535 (PMC8067831; doi:10.3390/genes12040535)
Supplement: Supplementary file 1 [file genes-12-00535-s001.pdf]

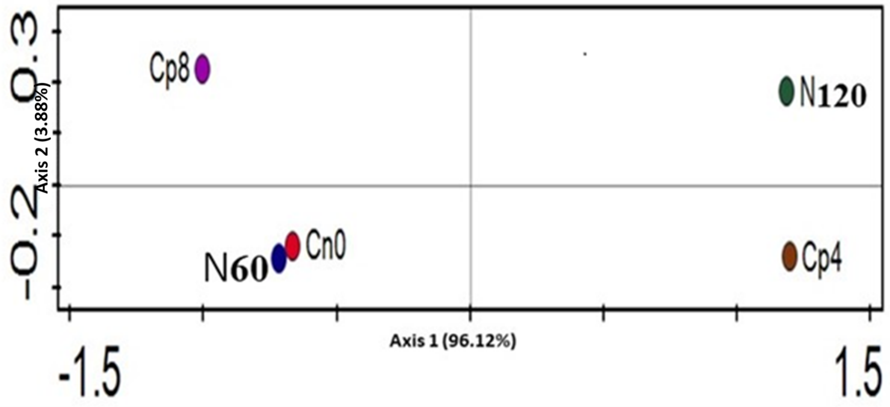

Fig. S1 PCoA (Principal Coordinate Analysis) showing the beta diversity between the maize rhizosphere soils at different fertilization regimes, Cn0 (control), N60 (60 kg/ha NPK), N120 (120 kg/ha NPK), Cp8 (8 tons/ha compost), and Cp4 (4 tons/ha compost manure). Adapted from Enebe and Babalola [24] with slight modification

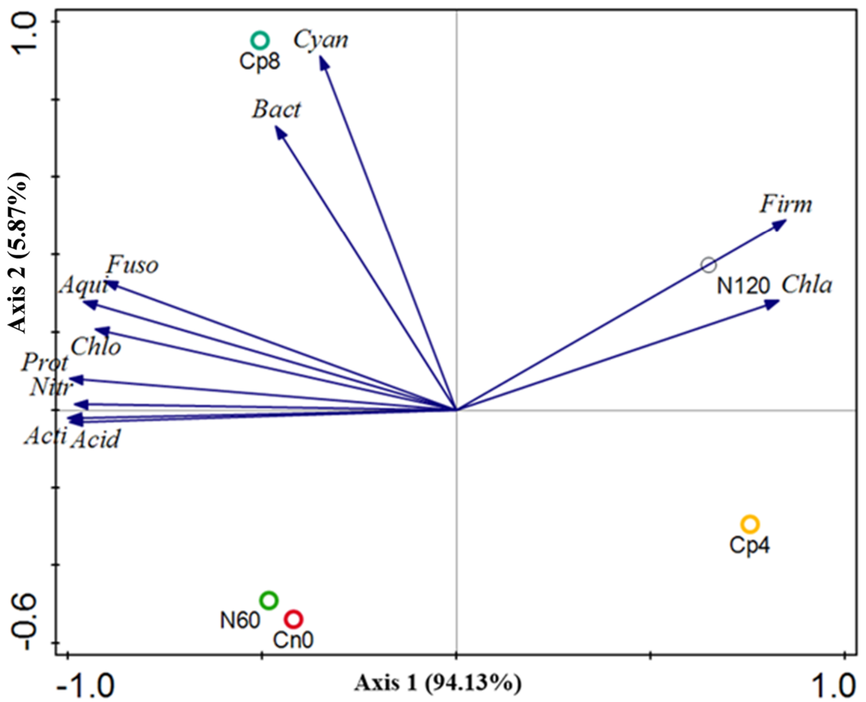

Fig. S2 PCA (principal component analysis) of the bacterial community structure associated with maize rhizosphere grown under different fertilization and unfertilized regimes (compost 8 tons/ha—Cp8, compost 4 tons/ha—Cp4, control—Cn0, 60 kg/ha inorganic fertilizer—N60, 120 kg/ha inorganic fertilizer—N120), showing treatments as the key factors influencing the structural shift and shape of bacterial community at the rhizosphere soil samples. The percentages represent the observed variations and the bacterial abbreviations are Acid (*Acidobacteria*), Acti (*Actinobacteria*), Aqui (*Aquificae*), Bact (*Bacteroidetes*), Cyan (*Cyanobacteria*), Chla (*Chlamydiae*), Chlo (*Chlorobi*), Firm (*Firmicutes*), Fuso

(*Fusobacteria*), Nitr (*Nitrospirae*), Prot (*Proteobacteria*). Adapted from Enebe and Babalola [24] with slight modification.
